# Bioimaging Probes Based on Magneto-Fluorescent Nanoparticles

**DOI:** 10.3390/pharmaceutics15020686

**Published:** 2023-02-17

**Authors:** Sayan Ganguly, Shlomo Margel

**Affiliations:** Department of Chemistry, Institute of Nanotechnology and Advanced Materials (BINA), Bar-Ilan University, Ramat-Gan 5290002, Israel

**Keywords:** nanoparticles, biomedical, magneto-fluorescent, multimodal imaging

## Abstract

Novel nanomaterials are of interest in biology, medicine, and imaging applications. Multimodal fluorescent-magnetic nanoparticles demand special attention because they have the potential to be employed as diagnostic and medication-delivery tools, which, in turn, might make it easier to diagnose and treat cancer, as well as a wide variety of other disorders. The most recent advancements in the development of magneto-fluorescent nanocomposites and their applications in the biomedical field are the primary focus of this review. We describe the most current developments in synthetic methodologies and methods for the fabrication of magneto-fluorescent nanocomposites. The primary applications of multimodal magneto-fluorescent nanoparticles in biomedicine, including biological imaging, cancer treatment, and drug administration, are covered in this article, and an overview of the future possibilities for these technologies is provided.

## 1. Introduction

There are several definitions of “imaging”. Most people consider imaging to be a subset of photography. These limitations are by no means present in scientific imaging [1]. Nuclear magnetic resonance (also known as magnetic resonance imaging; MRI) [2,3]; radioimaging with respective nuclides [4,5]; computed tomography (CT) [6,7]; positron emission tomography (PET) [8]; electrochemical imaging with rastering electrodes; mechanical methods, such as atomic force microscopy (AFM) [9]; and even more sophisticated scanning methods, such as laser ablation (ICP-MS) [10], mass spectrometry (MALDI) [11], and the like, can all be used to produce images. While many of these techniques are inapplicable to living systems or intact tissues due to their destructive nature or the substantial sample preparation they require, some are. Over the previous decade, several fluorescent microscopic instruments have been created [12,13], allowing for the visualization of biological functions at sizes ranging from the molecular to the cellular to the organ to the complete organism [14]. For instance, because of advances in super-resolution microscopy, scientists are now able to pinpoint the locations of individual molecules within cells with an accuracy greater than 10 nm [15]. This study aims to familiarize the reader with the wide variety of nanomaterials now available for fluorescence imaging and to help in choosing the best one for a given application.

**Figure 1:**
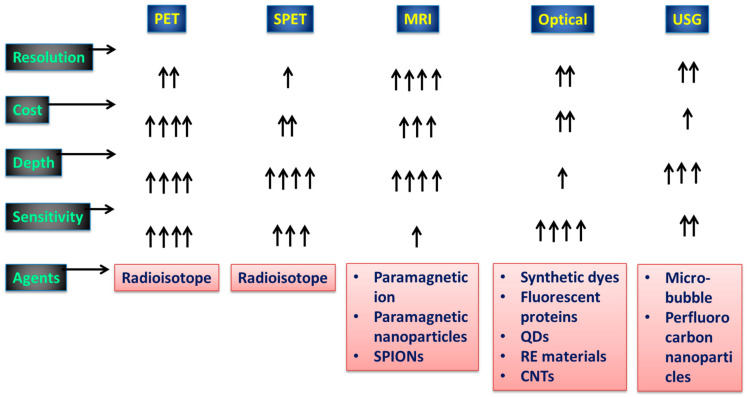
Aspects of diagnostic imaging systems employed in practice. The conventional diagnostic tools and their viabilities with respect to signal capturing are shown. A single arrow represents the minimum viability, and four arrows represents high viability.

A significant reduction in phototoxicity may be achieved with the utilization of light-sheet illumination, which also enables the rapid collection of sufficient data in a condensed amount of time to enable volumetric imaging [16]. The ability to see biological mechanisms has been revolutionized as a whole as a result of the combination of sensitive high-speed detectors, strong lasers, and speedy computers [17]. However, in order to provide an accurate representation of biological processes, these more sophisticated instruments require stronger reporting systems. Molecular imaging allows us to see and learn about individual molecules and tissues in living organisms [18,19]. Tracking cells after transplantation into a patient and subsequently detecting illnesses at an early stage before biochemical problems cause changes in anatomical structure is a rapidly growing field of study. Position emission tomography (PET), single photon emission computed tomography (SPECT), magnetic resonance imaging (MRI), optical imaging (OI), and ultrasonography (USG) are all examples of molecular imaging modalities that have recently become more useful in the diagnosis of a wide range of disorders [20,21,22]. Figure 1 details the capabilities of several of the most widely used medical imaging techniques. There is no perfect imaging modality that can offer all the necessary data because each modality has its own set of benefits and drawbacks [23]. As a result, it makes sense to mix imaging modalities in a way that maximizes the benefits of one while mitigating the drawbacks of the others. Nanoparticles (NPs) that are 100–10,000 orders of magnitude smaller than cells can be manipulated to cross cell membranes and aggregate in specific places. Owing to their distinct chemical and physical features, they can transport medications and imaging agents more effectively [24,25]. The use of NP-based multimodal imaging probes, which can be identified by multiple imaging modalities, has grown in importance because it paves the way for simultaneous multitargeting and monitoring, increasing diagnostic and therapeutic effects [26].

Many biological applications might benefit from the use of magnetic NPs, which possess a wide variety of desirable features. After the advent of X-ray technology, research into imaging agents took off, eventually expanding to encompass the radiolabeling of cells and tissues to better aid in the diagnosis and tracking of disease. Each product’s generic and commercial names were recorded, as was the company that received the first FDA approval (at a minimum, the year, and ideally the month and day). The FDA’s records often revealed the current distributors of products approved before or around the mid-1990s (and not necessarily the innovator organization if the product had changed hands as a result of licensing or acquisitive activities). Although much work went into reducing the toxicity of Collargol and other pioneering compounds, newer and safer contrast agents eventually supplanted colloidal silver [27]. Similarly, J. Edwards Burns introduced thallium nitrate as a contrast agent in 1915 [28]. Moses Swick made an important contribution to the development of urographic imaging [29]. The imaging method for breast cancer, especially melanoma staging, makes use of sulfur colloids [30]. The iron oxide nanoparticle Ferumoxytol (FerahemeTM, AMAG Pharmaceuticals) is coated with polyglucose sorbitol carboxymethylether and displays superparamagnetic characteristics [31]. Iron deficiency anemia (IDA) may be effectively and rapidly treated with ferumoxytol when it is injected intravenously (i.v.) at a dose of 510 mg. Gadolinium chelates are widely used because they are the safest MR contrast media. They are regarded as being less dangerous than nonionic iodinated contrast agents [32]. First-generation commercialized, FDA-approved ultrasound contrast agents (UCAs) were either nitrogen-based gases encased in peptide capsules (Albunex; Molecular Biosystems, Inc., San Diego, CA, USA) or powdered, hydrophilic particulate matter (Levovist; Bayer AG, Leverkusen, Germany) [33]. In order to aid doctors in ruling out Alzheimer’s disease as the only cause of cognitive decline, the Food and Drug Administration (FDA) has just green-lit a novel radiopharmaceutical agent. Individuals with cognitive impairment who are being tested for Alzheimer’s disease or other causes of cognitive decline may benefit from a positron-emission tomographic (PET) scan of the brain with an injection of florbetapir F18 (Amyvid, Eli Lilly, Indianapolis, IN, USA) [34]. When an external magnetic field is applied, magnetic NPs, for instance, can be directed to a single cell or organ of interest. Cancer hyperthermia treatment relies on the fact that an alternating magnetic field may be utilized to selectively heat the region in which magnetic particles are concentrated [35]. It is also possible to employ aqueous dispersions of superparamagnetic NPs as contrast agents for MRI (MRI) [36]. Combining magnetic and other features into a single nanocomposite structure paves the way for novel nanomaterials with interesting multimodal capabilities. Examples of potential biological applications include multimodal biological imaging, medical diagnostics, and drug-delivery systems, all of which might benefit from the creation of fluorescent-magnetic nanocomposites [37]. These nanocomposites have the potential to be used in multimodal tests for both in vitro and in vivo bioimaging techniques, including MRI and fluorescence microscopy [38]. They have both photodynamic and hyperthermic capabilities, making them potential bimodal medicines for cancer treatment [39]. The ability to readily regulate and monitor these nanocomposites using fluorescent or confocal microscopy and MRI makes cell tracking, cytometry, and magnetic separation fascinating new applications. Our analysis will be limited to the most up-to-date findings concerning magneto-fluorescent nanomaterials and a few of their therapeutic systems.

## 2. Types of Magnetic and Fluorescent Nanoparticles

### 2.1. Superparamagnetic Nanoparticles

**Figure 2:**
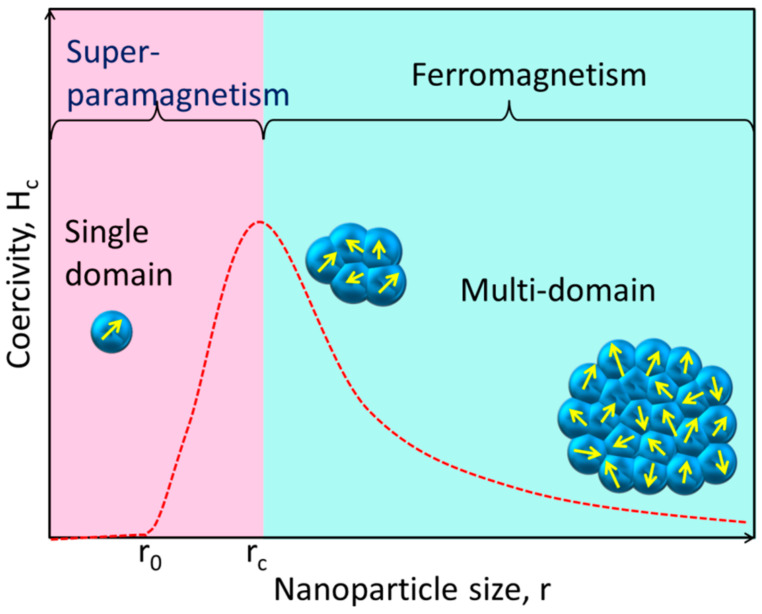
This simplified diagram shows how the magnetic coercivity (H_c_, the magnetic field needed to decrease the magnetization to zero) of a magnetic NP varies with its physical dimensions. As an NP is shrunk to the critical size (*r_c_*), the domain wall vanishes and the H_c_ rises. The NP reaches the superparamagnetic domain and exhibits zero coercivity if its size is further reduced to r_0_, where the thermal agitation energy is greater than the magnetic anisotropy energy and the magnetic moment of the NP varies freely.

Unlike their bulk counterparts, magnetic NPs have completely distinct magnetic properties. A huge number of magnetic domains make up the majority of ferromagnetic materials, and each domain has parallel magnetic moments that are isolated by domain walls [40]. Domain walls arise when magnetostatic energy and domain-wall energy are in equilibrium [41,42]. The domain-wall energy is responsible for the larger interfacial area between domains, and the magnetostatic energy grows proportionately with the volume of the material [43]. The energy required to create domain barriers is substantially more than the magnetostatic energy required to maintain a single-domain NP; hence, there is a critical value below which a stable single-domain NP can be established when the size of ferromagnetic materials is lowered to the nanoscale level [44]. As illustrated in Figure 2, the critical size (*r_c_*) of an NP may be stated according to Equation (1) when the magnetostatic energy is equal to the domain-wall energy and the NP is transitioning from the multidomain state to the single-domain state [45].

(1) $$r_{c}=18\frac{\sqrt{AK_{eff}}}{\mu_{0}M^{2}}$$

where *A* represents the exchange constant, *K_eff_* is the anisotropy constant, 0 is the vacuum permeability, and *M* is the saturation magnetization. The critical diameter of magnetic NPs is generally between 10 and 100 nm.

The magnetic spins of ferromagnetic NPs are linked and parallel-aligned inside a single domain, as was indicated before. When the magnetic moments are kept in a single domain of ferromagnetic NPs, the magnetic anisotropy energy [46] (Δ*E*) is calculated as follows:

(2) $$\Delta E=K_{eff}V$$

**Figure 3:**
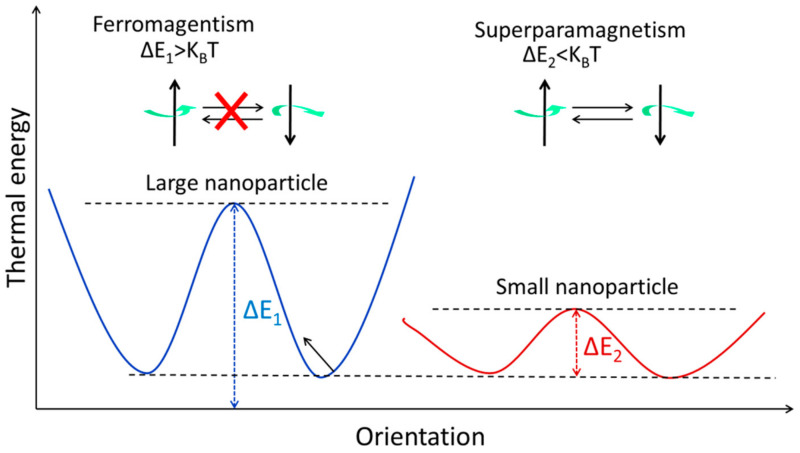
The energy levels of magnetic NPs with varying magnetic spin orientations. When it comes to stopping the magnetization from rotating, thermal energy (K_B_T) is the limiting factor. Both ferromagnetism and superparamagnetism may be seen in the large and small NPs, respectively.

Figure 3 shows how to calculate the volume of an NP using Equation (2).

### 2.2. Fluorescent Nanoparticles

Over the course of the last ten years, research and development efforts have been heavily concentrated on fluorescent NPs [37,47,48,49,50,51]. These NPs include semiconductor NPs (quantum dots), metal NPs, silica NPs, polymer NPs, and many more [52,53,54]. In comparison to traditional fluorescent organic dyes, fluorescent NPs exhibit a more brilliant fluorescence, increased photostability, and enhanced biocompatibility. In addition, the NPs have a distinct set of chemical and optical characteristics [55,56]. Several different kinds of fluorescent NPs have emerged for bioimaging over the past few decades. These include organic-dye-doped silica NPs, organic polymer NPs, metallic NPs, carbon-based NPs (nanotubes and nanodots) [57], quantum dots (QDs) [58,59,60], and lanthanide-doped upconverting nanoparticles (UCNPs) [61,62,63]. In terms of their composition, fluorescent NPs employed in bioimaging may be roughly divided into two categories: organic and inorganic NPs. In order to be considered among the best fluorescent probes for imaging, fluorescent NPs should generally meet the following characteristics: high signal-to-background ratio, big strokes to prevent self-quenching, excellent stability under physiological settings, little cytotoxicity, and minimum disruption of biological activities, to name only a few. They also have limited fluorescence durations, typically around 10^−9^ s, which is insufficient for efficiently separating short-lived fluorescence interference from dispersed excitation light.

Optical biosensors based on fluorescence may be divided into two categories: those that use downconversion fluorescence and those that use upconversion fluorescence. Downconversion fluorescence, the foundation of most existing optical biosensors, works by transforming the energy of light, often in the ultraviolet to visible light range, into a more usable form. These probes excel in chemical, photochemical, and thermal stability, and their long fluorescence lifetimes make them ideal for biosensing applications. They are vulnerable to photobleaching and blinking, and their overlapping excitation and emission spectra are a challenge for multiplexing applications [64]. In addition, biological samples include endogenous fluorophores, such as hemoglobin, which strongly absorb and scatter light below 700 nm, resulting in a high background fluorescence that can only penetrate biological media superficially. These limitations of downconversion-based biosensors have severely stymied their use in biosensing and bioimaging. Nanoparticles based on upconversion fluorescence (UCNPs) have significant potential for use in biomedical settings due to their ability to convert near-infrared (NIR) light into visible light [65]. Due to its providing an optically clear window onto biological tissues, NIR light has attracted a lot of attention as an excitation source for biosensing and bioimaging in recent years [66].

### 2.3. Types of Noninvasive Imaging

Recently, a variety of noninvasive optical imaging techniques, including computed tomography (CT), magnetic resonance (MR), positron emission tomography (PET), single-photon emission CT (SPECT), ultrasound (US), and optical imaging (OI), as well as their variants and subcategories, have been described [67,68]. Each one is distinct from the others in respect of resolution and sensitivity complexity, the length of time required to obtain data, and cost. The selection of an imaging modality is largely determined by the precise question that needs answering, and various imaging modalities are often complimentary rather than in competition. Medical imaging with magnetic resonance (MRI) is another technique that has expanded greatly in recent years. MRI is particularly useful for diagnosing conditions affecting soft tissues [69]. Furthermore, the drawbacks of each approach, such as MRI’s low sensitivity and OI’s lack of anatomical background information, cancel each other out.

### 2.4. Magnetic Resonance Imaging (MRI)

**Figure 4:**
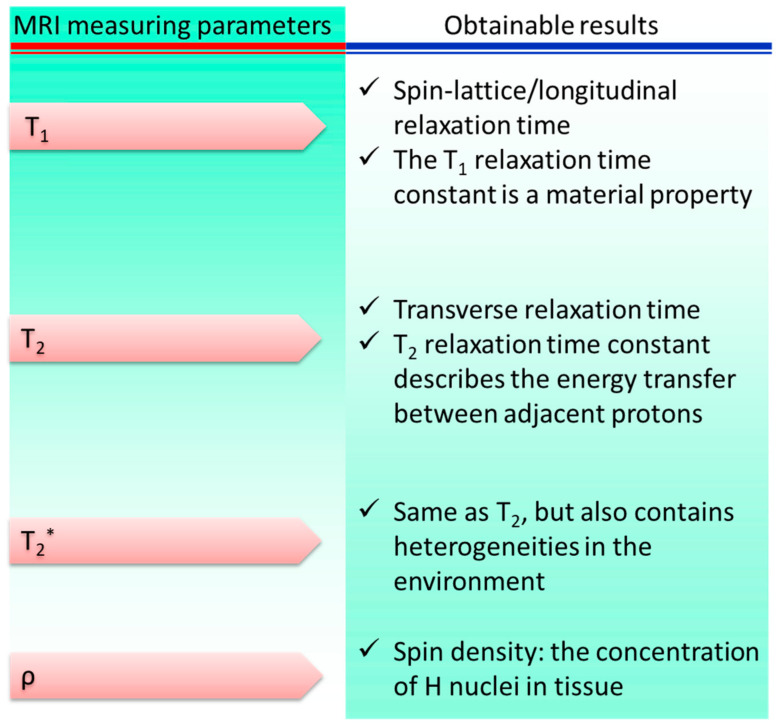
Parameters of magnetic resonance imaging: sources of signal variation. * represents a separate variable.

The MRI method was developed from the foundations of nuclear magnetic resonance. NMR signals obtained from hydrogen nuclei placed in various physiological contexts throughout an organism are used to create tissue contrasts, which are then used for diagnostic purposes. The frequencies at which nuclear spins resonate when a specimen is immersed in a homogeneous, static magnetic field are proportional to the strength of the magnetic field. Specimens are activated by a radiofrequency pulse at their resonance frequency in order to alter their net magnetization once they have established an equilibrium magnetization. Three-dimensional pictures of the body are constructed by measuring the variations in generated electromagnetic signals in the presence of linear field gradients. MRI is a powerful diagnostic tool for detecting lesions in the brain and spinal cord due to its high tissue specificity. Figure 4 shows the main contributors to the overall signal and contrast levels obtained from a sample.

### 2.5. Computed Tomography (CT)

Over the past decade, there has been a meteoric surge in the number of papers published on the use of microcomputed tomography (CT) imaging in preclinical in vivo research [70,71]. Better spatial and temporal resolution has enabled researchers to acquire more precise anatomical pictures of small animals and track the development of illnesses in small-animal models. Although organs and tumors are not easily visible on CT images without the use of iodinated contrast agents, CT has poor soft-tissue contrast for malignancies and surrounding tissues [72,73,74]. At first, CT had great spatial resolution but low contrast in soft tissues. The noninvasive examination of high-contrast structures, such as bones and implants, was hence the primary emphasis of the earliest publications on the utilization of CT. There have been significant increases in the temporal and geometrical resolutions, as well as the readout speeds, of X-ray detectors as technology has developed. A µCT method with a spatial resolution of 1–100 µm is denoted as µCT [75]. µCT has the potential to supplant the laborious serial staining procedures needed for the histomorphometric examination of thin slices, and it might also be used to conduct longitudinal in vivo investigations in tiny animals. Given CT’s short imaging duration and great spatial resolution, it can be utilized to examine lung cancers and bone metastases. µCT has been employed in high-throughput phenotyping methods for a large number of transgenic mice, allowing for the detection of gross defects. To examine cell trafficking, tumor development, and response to treatment in vivo, µCT images showing tumor structural features were coregistered with bioluminescence pictures. This approach to image processing has the potential to be employed in evaluating hematological reconstitution after bone-marrow transplantation. CT is useful because of the high spatial resolution (12–50 µm) that is required to observe minute anatomical features. Functional imaging methods can be used with CT to reveal metabolic and dynamic details. Comparisons of in vivo trabecular structures and mineralization densities between mice strains were made in osseous disease investigations to account for any variations in experimental design and data collection [76]. Similar to CT angiography (CTA) in humans, contrast-agent injection is required for in vivo examination of vascular systems using CT in small animals. Vessel analysis in rats after sacrifice [13] was possible with the older, somewhat slow CT scanners, with scanning periods extending up to hours, employing perfusion with radiopaque polymerizing chemicals [77] or shock freezing of the contrast-agent-perfused material [78].

## 3. Synthesis and Fabrication of Magneto-Fluorescent Nanoparticles

Nanotechnology has grown rapidly over the past few decades as a new discipline that bridges previously separate scientific disciplines, such as biology, medicine, chemistry, materials engineering, quantum mechanics, and electronics. The benefits of nanomaterials, such as their high surface-to-volume ratios, unique optical characteristics, and nanoscale physical phenomena, have led to their widespread usage in scientific study and medical practice. Superparamagnetism at room temperature is a defining characteristic of MNPs (e.g., Fe_3_O_4_, γ-Fe_2_O_3_, or a combination of the two) when their size is small enough [79]. Molecular nanoparticles (MNPs) have the potential to be used in ferrofluid technology [80] and heterogeneous catalysis due to their extremely high surface-to-volume ratios and in the cleanup of polluted or contaminated environmental media [81]. Furthermore, it has recently been considered that MNPs might be used for a variety of biological applications (magnetic contrast agents, hyperthermia agents, magnetic vectors for drug administration, etc.). While MNPs have great potential for biomedical applications, they must first meet a number of requirements that are often contradictory. These include: (a) extremely low toxicity to the human body; (b) outstanding magnetic properties; (c) a relatively narrow size distribution; and (d) the ability to have their surfaces easily modified (through coating) in order to allow their functionalization for particular bioagents. Since magnetite (Fe_3_O_4_) and maghemite (γ-Fe_2_O_3_) NPs have been shown to be biocompatible, they are good choices to meet the first two requirements [79]. Additionally, MNPs with high stability, biocompatibility, and low toxicity may be created and employed in a wide range of biomedical applications after being modified by functional components [82].

The creation of specific magneto-fluorescent NPs has attracted a lot of attention in recent years. Although these nanocomposites have a wide range of compositions and morphologies, we have categorized the preparation techniques into two types of synthetic strategy: the coupling approach and the encapsulation method, based on reports from a variety of sources. Table 1 lists several common methods for synthesizing composite NPs and the various ways they might be employed.

**Figure 5:**
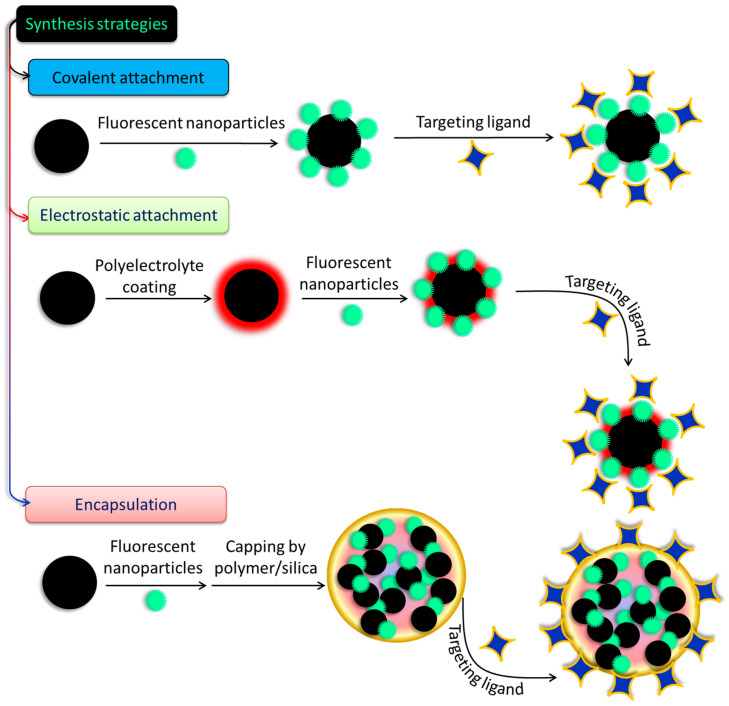
Different approaches to prepare fluorescent-nanoparticle-decorated magnetic nanostructures.

There have been few attempts made to construct hybrid systems, despite the rising interest in and the relevance of SPIONs and SiQDs to the fabrication of biodegradable and biocompatible nanoprobes for biomedical use. Conjugation of the two systems and studies of their conjugated characteristics are scarce, and obstacles such as charge or energy transfer processes and iron oxide’s high absorption in the visible spectrum make even their simple fabrication by quenching QD/SiQDs PL difficult [100]. Long interparticular spacers are usually employed to avoid such disadvantages and ensure the flourishing of both luminous and magnetic characteristics [101]. Covalent binding and electrostatic absorption are used to couple the magnetic and fluorescent species shown in Figure 5 after they have been synthesized and functionalized independently. In this procedure, the coupling strategy for magnetic and fluorescent NPs is determined by the modifications made to their surfaces. Thiol, carboxyl, and amino groups are all examples of functional moieties that may be found in the coupling agents [102]. The formation of core–shell structures on the surfaces of magnetic materials often involves loading fluorescent elements onto the top of a magnetic material by either bond formation or charge attraction. After that, conjugation of specific ligands onto the surfaces of the magneto-fluorescent NPs is often performed. In another method, which is illustrated in Figure 5, the cores of new composite micro- or nanospheres are formed by encasing prefabricated fluorescent NPs and magnetic NPs in various materials, such as silica or polymer beads, protein, chitosan, and liposomes. This results in the formation of new NPs. In order to create these composite nanospheres, two different approaches have been utilized. These materials give the NPs favorable features, such as strong biocompatibility and stability and facile functionalization, to enable the inclusion of ligands so that the NPs may be selectively targeted physiologically. These materials also make the incorporation of ligands easier [103]. Since the existing coupling techniques are straightforward and provide a number of important benefits, a wide variety of magneto-fluorescent NPs have been fabricated and put to use in a variety of subfields of biomedicine. However, the stability of NPs generated in this manner frequently shifts in response to varied settings; as a result, their applications in biomedicine are restricted. When compared to magneto-fluorescent NPs created using the coupling approach, those prepared using the encapsulation method provide a number of benefits, including the ones listed below:

(a) NPs can be covalently modified with diverse targeted ligands using the surface functionalities of silica or polymer nanospheres without a stable structure.
(b) However, controlling the proportion of MNPs to luminescent NPs is difficult, making the production of well-dispersed, homogeneous, multimodal NPs complicated.
(c) Additionally, the outer layer of silica or polymer, which may serve as a screen, may play a role in preventing unwanted particles from entering.

## 4. Magento-Fluorescent Nanobioprobe for Cancer Targeting

**Figure 6:**
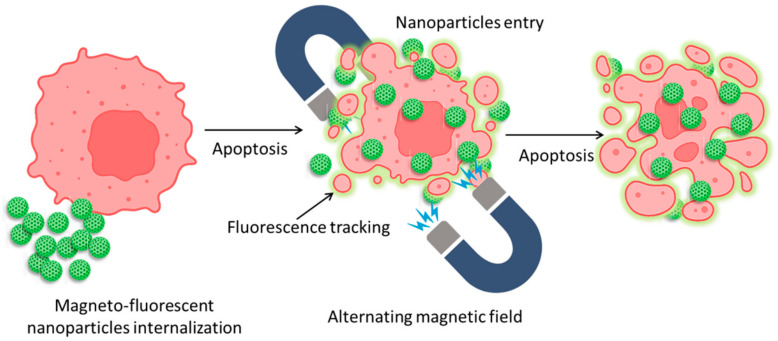
Mechanism of magnetic nanoparticle internalization into cancer cells and their tracking and apoptotic behavior against an alternating magnetic field.

The health sector can benefit greatly from fluorescent and magnetic materials. Due to their unique optical and magnetic capabilities, typical materials such as quantum dots (QDs) and iron-oxide NPs have attracted the attention of scientists for decades. Nanomaterials such as quantum dots (QDs) have the potential to revolutionize in vitro biolabeling, in vivo targeting, and imaging thanks to their unique combination of broad and continuous absorption, narrow and symmetric fluorescence emission, and intense and steady fluorescence. Bioseparation, immobilization of cells and enzymes, medication administration, magnetic resonance imaging, and thermotherapy are just a few of the many applications of magnetic nanoparticles. QDs and magnetic nanoparticles have been found to coexist in nanocomposites in recent years. Macrophages and related immune cells have been intensively explored for insights into the internalization pathways of synthetic NPs, such as superparamagnetic iron oxide nanoparticles (SPIONs) and gold NPs. Upon internalization of SPIONs, multivesicular bodies are formed in the cytoplasm via a time-dependent vesicle-bound route. It is generally agreed that some internalized SPIONs are degraded in lysosomes, while others are released into the cytoplasmic compartments for continued metabolism [104]. Previous research has shown that NPs such as gold NPs are subject to varying degrees of absorption by HeLa cells depending on various factors, including particle size and shape [105]. A simplified graphical illustration has been given in Figure 6 for easy visualization.

**Figure 7:**
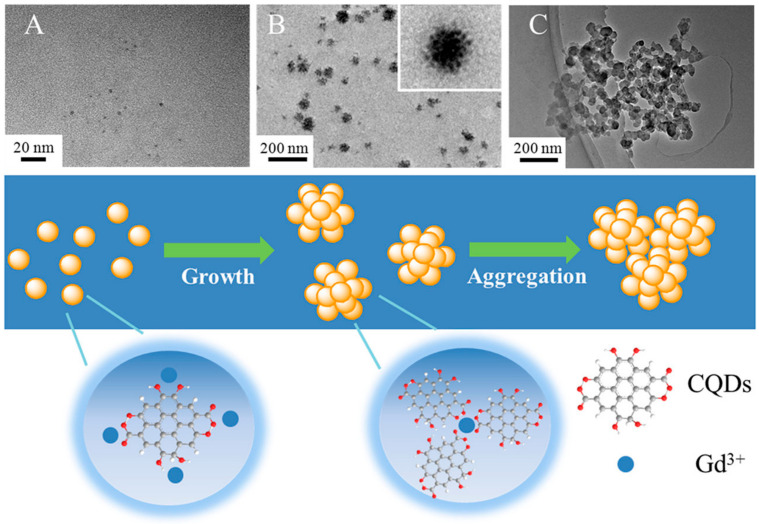
(**A**) TEM photos of the hydrothermal products after varied incubation durations, including (**A**) 1 h, (**B**) 3 h, and (**C**) 5 h, as well as a schematic illustration of the process of Gd–CQD synthesis [113].

**Figure 8:**
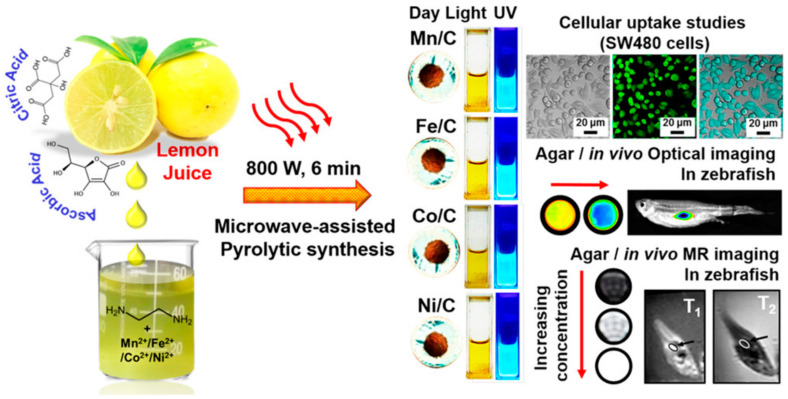
Microwave-assisted pyrolytic synthesis of EDA-functionalized TMCDs as possible nanoprobes for magneto-fluorescent bioimaging depicted schematically [119].

Quantum dots (QDs) were immobilized on the surface of superparamagnetic polystyrene nanospheres, and the novel combination of fluorescence emission and hyperthermia engineered in these nanocomposites is expected to be used in therapeutic trials for concurrent in vivo imaging and local therapy via hyperthermia [106]. Similarly, in another study, CdTe/CdS quantum dots (QDs) and Fe_3_O_4_ NPs were encapsulated in silica-coated magnetic polystyrene nanospheres (MPNs) [107]. Although the bioconjugates were useful in identifying and sorting cancer cells, they were unable to perform these functions in the case of K562 cells because they lacked surface expression of human epidermal growth factor receptor (EGFR). By using a direct amide coupling procedure, iron oxide nanoparticles and CdS quantum dots were successfully synthesized to form a multifunctional hybrid nanocomposite material [108]. These NPs were further coupled with folic acid to test their potential as a molecular imaging probe. Cell uptake and cytotoxicity experiments were performed on normal mouse splenocytes, C6 rat glioma cells, and A549 human lung adenocarcinoma epithelial cells. C6 cell green fluorescence emission demonstrated uptake of nanoparticles. Research using Prussian blue staining has hinted at the existence of iron oxide within cells. To add to this, it was shown that folic acid-conjugated nanocomposites were considerably hazardous to C6 cells only after 48 h, but not to A549 cells or splenocytes. Chitosan-encapsulated NPs of iron oxide (for use as MRI contrast agents), cadmium sulfide (for use as a fluorescence probe), and podophyllotoxin (for use as an anticancer medication) were produced and described as multifunctional hybrid nanocomposite materials [109]. KB, C6, and A549 cancer cell lines were used to test the efficacy of these nanocomposites in combating various forms of the disease. The existence of these nanocomposites in KB and C6 cells, but not A549 cells, was verified by fluorescence imaging and Perl’s Prussian blue staining. Experiments on cytotoxicity demonstrated that the nanocomposites covered with biopolymers were only slightly harmful to cancer cells. For targeted tumor imaging, NPs with strong near-infrared (NIR) fluorescence were produced for use in T_1_- and T_2_-weighted MR imaging. Wang et al. combined T_1_- and T_2_-weighted magnetic resonance imaging (MRI) with fluorescence imaging of malignancies by incorporating ultrasmall Fe_3_O_4_ NPs (2–3 nm) with an NIR-emitting semiconducting polymer [110]. Multimodal fluorescent magnetic nanoparticles (FMNPs) with folic acid functionalization glow brightly under fluorescent light and relax slowly. In a different study, the thiol-induced assembly of magneto-fluorescent nanoparticles mimicking hydrangea flowers was described [111]. Thiol-metal bonding and surface functionalization allow for a straightforward strategy for creating magneto-fluorescent Fe_3_O_4_-SH@QD NPs which have the form of flowers like hydrangeas. MRI and biolabels, targeting and photodynamic treatment, cell tracking and separation, amongst other technologies, might all benefit from magneto-fluorescent Fe_3_O_4_-SH@QD NPs’ efficient fluorescence, superparamagnetism at room temperature, and fast reaction to an external field. For fluorescence and magnetic resonance imaging of cancer cells, setua et al. developed a nanobioprobe based on doped Y_2_O_3_ nanocrystals [112]. Up to this point, the vast majority of research efforts have been focused on creating C-dots doped with nonmetals. Furthermore, there have been very few reports of investigations concerning the production and usage of heavy-metal-doped C-dots. Gadolinium-doped C-dots, also known as Gd-CQDs, are dual-fluorescence and MRI probes that were recently synthesized by Yang Xu et al. [113]. Both in vitro and in vivo testing demonstrated that they have low toxicity levels and are highly biocompatible. Due to the large amount of Gd present in Gd–CQDs and their hydrophilicity, it was shown that these particles had a greater MR response than gadopentetic acid dimeglumine (Gd–DTPA) (Figure 7). The authors discovered that Gd–CQDs dispersed themselves in tissues in a heterogeneous manner: some of them penetrated the tissue cells, while others were identified less often in the extracellular matrix. In order to detect Fe^3+^ ions, Quan Xu and colleagues created Cu-doped C-dots to use as fluorescent-sensing probes [114]. Recent research carried out by our team has resulted in the successful development of Ag/C-dot and Au/C-dot nanohybrids derived from lemon extract for the purpose of cancer cellular imaging [115]. To begin with, metal-oxide NPs, such as iron-oxide (Fe_2_O_3_/Fe_3_O_4_), manganese dioxide (MnO), yttrium dioxide (Gd_2_O_3_), and dysprosium dioxide (Dy_2_O_3_), were used [116,117,118]. Pakkath et al. used lemon extract and an efficient, one-pot, microwave-assisted pyrolysis process (Figure 8) to produce transition-metal-ion-doped C-dots (TMCDs) containing Mn^2+^, Fe^2+^, Co^2+^, and Ni^2+^ within 6 min [119].

**Figure 9:**
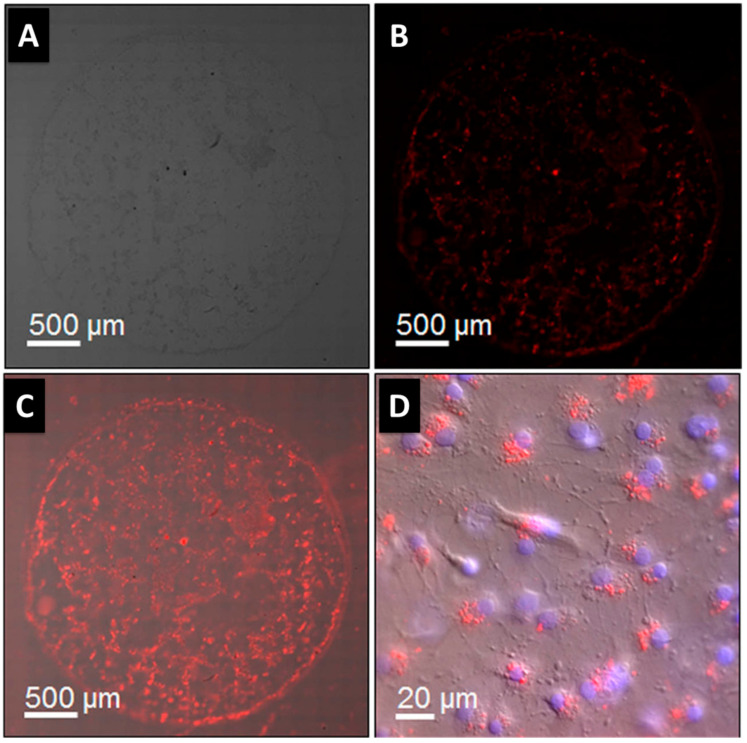
(**A**) Bright-field picture showing all cells; (**B**) red fluorescence image revealing QD in nanoparticles, which are specifically localized using a Rhodamine filter; (**C**) merged image; (**D**) magnification of (**C**) showing few individual cells. Hoechst staining marks cell nuclei blue. Nanoparticles (red dots) near nuclei indicate cell localization [120].

The success of these magneto-fluorescent NPs will depend heavily on their sizes, which must be programmable, the magnetic and optical signals, their long-term stability, and the variety of surface functionalities. The MRI contrast agent is encapsulated in a fluorescent quantum dot (QD) core that is enclosed inside a hollow magnetic shell, as shown by pahari et al. [120]. As can be observed in Figure 9A–D, a fluorescence quantum efficiency of roughly 16% (measured in buffered aqueous media) is adequate for optical microscopy imaging of 3D cell growth. Images taken with a wide-field optical microscope revealed a single drop of 3D cell culture on a Matrigel scaffold, originating from mouse cerebral tissue, on a 32 mm cell-culture dish. CdSe@CdS@Fe_2_O_3_ nanoparticle markers were added to the Sprague–Dawley rat embryonic cells 6 days before harvesting the cells at day 13 in vitro. Red fluorescent NPs (620 nm peak) were likely to be localized inside the cells, as they were found in close proximity to the nuclei.

High-efficiency cellular imaging was achieved by preparing and using new fluorescent/magnetic NPs [121]. The nanoparticles covered in modified chitosan had a magnetic oxide core and a fluorescent dye linked to it through covalent bonds. We tested how well the NPs worked at marking cancer cells. Flow cytometry and magnetic resonance imaging both showed that the nanoparticles had a strong affinity for cells. The findings demonstrated that the nanoparticles’ efficacy in labeling cells was sensitive to both the length of incubation and the concentration of the nanoparticles used.

It is becoming increasingly obvious that techniques for identifying multiple key targets in size-limited clinical specimens will be essential in order to assess the temporal and spatial conditions of signal transduction networks and to realize the goal of personalized medicine. The molecular diversity of human cells, especially cancer cells, is quite high. Western blotting, flow cytometry, immunofluorescence imaging, and immunohistochemistry require extraordinarily high cell densities, cannot be multiplexed, and have low throughputs. As a result, these approaches are restricted in terms of the quantity of information that can be retrieved from clinical specimens. Improved intracellular biomarker sensing using nanoparticles was reported by Haun et al.; the method used is based on bio-orthogonal chemistry [122]. They also demonstrated that this method could detect protein biomarkers and phosphoprotein signal mediators in the cytosol and nucleus, respectively, using either magnetic or fluorescence modalities. In order to enclose highly ferrimagnetic nanoparticles and ZnS/InP quantum dots, Song et al. described the use of an amphiphilic block copolymer containing a flowable hydrophobic chain [123]. The uniform diameter of the resulting ferrimagnetic fluorescent micelle (FMFM) was around 180 nm. Common poly(D,L-lactide) (PLA)-based amphiphilic block copolymer with a stiff hydrophobic chain unavoidably led to greater aggregation (400 nm in diameter), which was easily removed by the reticuloendothelial system (RES). Long-term colloidal stability in the flowable FMFM was seen within one month, and the necessary fluorescent stability was achieved within 84 h. Iron-based nanoparticles (IBNs) are ideal because they are biocompatible and may be directed at a tumor by a variety of methods (passive targeting, active targeting, or magnetic targeting). When necessary, IBNs can also be combined with other well-known fluorescent substances, such as dyes, ICG that has been authorized for therapeutic use, fluorescent proteins, and quantum dots. Depending on whether or not IBNs are paired with a fluorescent substance, they may also be stimulated and identified by means of existing optical techniques that rely on scattering or fluorescence processes. Systems that combine IBNs with optical techniques are versatile, allowing for several approaches to tumor detection. Such methods can also identify solitary tumor cells by combining IBNs with near-field scanning optical, dark-field, confocal, and super-resolution microscopy and recognize interactive manifestations, such as the permeation of a chromophore substance in an organism by utilizing photoluminescence lifetime imaging and fluorescence correlation spectroscopy. There is significant promise in the biological sciences for site-specific multimodal nanoplatforms with fluorescent-magnetic characteristics. By linking the human holo-transferrin to an optically and magnetically active bimodal nanosystem composed of quantum dots and iron oxide nanoparticles, Filho et al. created a multimodal nanoprobe (BNP-Tf) (Tf) [124]. There are several types of magneto-fluorescence for multimodal imaging and drug-delivery purposes, as shown in Table 2. The new BNP-Tf nanoplatform was effective in labeling the TfR at doses that did not produce any substantial toxic impact in HeLa cells for at least 24 h and remained active for at least two months. Using MRI, flow cytometry, and fluorescence microscopy, we showed that HeLa cells tagged specifically with BNP-Tf not only generated a strong fluorescence but also displayed a high r_2_/r_1_ relaxivity ratio, making it potentially an appealing probe for obtaining fluorescence and T_2_-weighted MR images. Enumerating and analyzing CTCs in circulation has become a powerful tool in cancer diagnosis and prognosis. However, for accurate and sensitive CTC detection, it is a major difficulty to successfully harvest lowly abundant CTCs with high purity from blood samples in a quick and high-throughput way. To rapidly magnetically isolate CTCs from human blood with high capture efficiency and purity approaching 80%, a novel class of DNA-templated magnetic nanoparticle-quantum dot (QD)-aptamer copolymers (MQAPs) were designed. QD photoluminescence (PL) is used to concurrently profile the phenotypes of CTCs at the single-cell level. These MQAPs are built via a hybridization chain process to boost the magnetic response, increase the binding selectivity for target cells over background cells, and make the QD PL in the ensemble extremely bright, allowing for single-cell identification. Nonspecific binding does not occur on MQAPs, ensuring that captured cells are as pure as possible. For the purpose of magnetically isolating rare cancer cells, li et al. produced a new class of MNP-QD-aptamer copolymers with high CE and CP that are advantageous for practical applications. These MQAPs are ideal for isolating and identifying CTCs in challenging environments thanks to their linear compact construction, strong magnetic sensitivity, excellent selectivity, and high PL intensity [125]. This method not only has excellent capture purity, but also identifies CTC phenotypes without the need for any further fixation or permeabilization processes. It was possible to successfully release the MQAPs from the cells while the cells themselves remained alive upon isolation. This provides new opportunities for the bioinspired design of tunable inorganic magneto-fluorescent materials for use in healthcare. Cancer molecular imaging (MI) is an emerging area of diagnostic imaging that offers new ways to study cancer biology in living organisms. In an effort to improve signal and/or contrast, binding avidity, and targeting specificity in the early detection of cancer, a wide range of targeted nanoprobes (NPs) have been created. Cancer cells differ from normal cells in their overexpression patterns of folate receptors (FRs) on the cell surface. Therefore, there has been a lot of interest in using folic acid (FA) or folate-conjugated NPs as diagnostic agents and treatments and even their combination usage as theranostics to target FR-overexpressing tumor cells. Owing to the strong binding between the ligand and its receptor, FR-specific MI methods have a number of advantages. Magnetic resonance imaging (MRI), computed tomography (CT), optical and nuclear imaging (ONI), and ultrasonography (US) are just a few of the clinical imaging modalities that may be tailored for use with NPs.

## 5. Magento-Fluorescent Nanobioprobe for MRI

**Figure 10:**
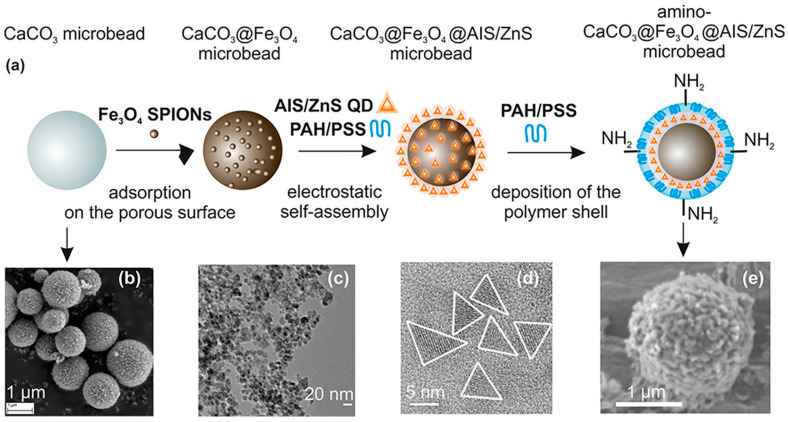
(**a**) Illustration of multistep synthesis of aminated magneto-fluorescent CaCO_3_ microbeads and (**b**–**e**) SEM and TEM pictures of building blocks: (**b**) CaCO_3_ spherical microparticles, (**c**) Fe_3_O_4_ SPIONs capped with PSS, (**d**) AIS/ZnS QDs stabilized with MPA, and (**e**) CaCO_3_@Fe_3_O_4_@AIS/ZnS microbeads containing amino surface groups for further bioconjugation [158].

Magneto-fluorescent particles are known to be promising new materials for cutting-edge uses. However, it has been difficult to synthesize magneto-fluorescent nanomaterials with desirable characteristics, such as uniform and adjustable size, high-magnetic-content loading, maximum fluorophore coverage at the surface, and flexible surface functioning. An easy method was revealed by chem et al. for combining magnetic nanoparticles with fluorescent quantum dots to create colloidal magneto-fluorescent supernanoparticles [131]. The core of these supernanoparticles is made up of tightly packed magnetic nanoparticles, while the shell is made up entirely of fluorescent quantum dots. High colloidal stability and biocompatibility, as well as adaptable surface functionality, are all provided by a thin layer of silica coating. Following surface pegylation, we showed that these silica-coated magneto-fluorescent supernanoparticles may be magnetically controlled inside live cells while being optically monitored. Quantum dots (QDs) doped with gadolinium paramagnetic ions have been identified as promising materials for fluorescence and magnetic-field-driven aquatic applications. Some intensely fluorescent Gd-doped AgInS2 QDs were supposedly synthesized at Gd molar ratios of 18–20 [156]. The new materials showed improved PL characteristics at greater Gd loadings than state-of-the-art materials, which showed poor PL features at higher Gd loadings. Aging, and the subsequent formation of a smaller particle size distribution, was linked to an improvement in PL characteristics. Increased medication delivery, water purification, and pollution monitoring are only some of the potential uses of this material, as demonstrated by the increased and sustained fluorescence at increasing magnetic Gd loadings. In order to meet the demand for magneto-fluorescent properties in a single unit, core–shell multifunctional nanostructures were produced [157]. High stability and integrity of the core and shell were achieved in the development of CdS quantum dots (QDs) on the surface of SrFe 12O19 nanoparticle cores, as evidenced by UV-visible spectroscopy, photoluminescence spectroscopy, VSM, and FTIR. Optical investigations can rule out the presence of flaws or core–shell structures and reveal comprehensive data on the emergence of any new phase at the interface. For nanostructures with core–shell geometries, the single magnetic domain structure is evinced by a small Stoner–Wohlfarth value. The goal of achieving anisotropy in only one direction, or “uniaxiality”, was successfully met. The use of water in the synthesis broadens the potential range of uses for these nanostructures, especially in biology. Chitosan-encapsulated NPs of iron oxide (as MRI contrast agents), cadmium sulfide (as a fluorescent probe), and podophyllotoxin (as an anticancer medicine) were produced and described to create a multifunctional hybrid nanocomposite material [109]. Human oral cancer (KB) cells, rat glioma (C6) cells, and human lung adenocarcinoma (A549) cells were used for the in vitro research. Both fluorescence imaging and Perl’s Prussian blue staining revealed the intracellular localization of these nanocomposites. The feasibility of these nanocomposites as dual-mode imaging probes was demonstrated by in vivo fluorescence imaging and Prussian blue staining investigations. Deposition of these nanocomposites in lung cells was seen in Wistar rat model biodistribution and toxicity tests, with no obvious ill effects on other essential organs. Cd-free Ag-In-S ternary quantum dots (t-QDs) with fluorescence lifetimes (LTs) of several hundred nanoseconds (ns) were paired with superparamagnetic Fe_3_O_4_ nanoparticles (SPIONs) and mesoporous CaCO_3_ microbeads (Figure 10) to create a magneto-fluorescent bead platform, as described by Martynenko et al. [158], who also evaluated the feasibility of using these magneto-fluorescent microbeads as magneto-fluorescent carriers with distinct LT signatures for time-resolved flow cytometry in the biological sciences and biotechnology (LT-FCM). For a recently designed flow cytometer using photon counting detection and polymeric carrier beads doped with various organic dyes, we recently proved this encoding technique.

**Figure 11:**
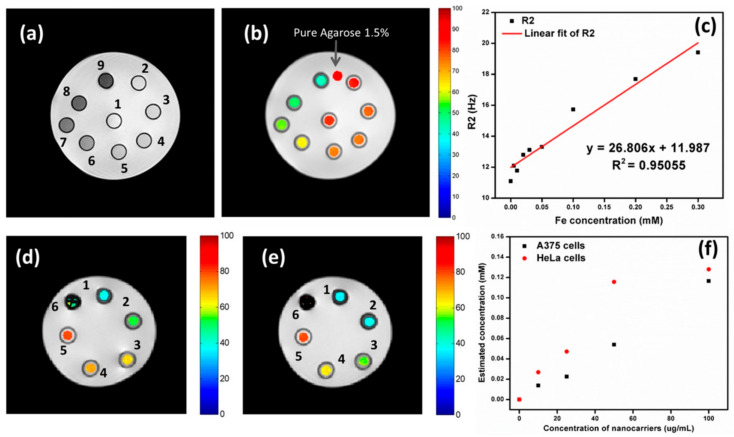
MRI (**a**) with a T2 weighting and (**b**) with a T2 map (ms) superimposed in color. MFCSNPs-FA-CHI-5FU nanocarrier concentrations are shown in part as numbers (1–9) that correspond to areas representing miniature phantom tubes. (**c**) Different concentrations of MFCSNPs in agarose (1.5%) were used to create phantoms, as detailed in the Experimental section. Fe concentration against R2 plot for MFCSNPs-FA-CHI-5FU nanocarriers (red line represents a linear fitting). MRI scans with color overlays of (**d**) A375 and (**e**) HeLa cells. Small phantom tubes containing A375 and HeLa cells were treated with varying quantities of MFCSNPs-FA-CHI-5FU nanocarriers (0–200 g/mL), as shown by the numbers (0 refers to cells only and 2–6 correspond to various concentrations of nanocarriers (0–200 g/mL)). (**f**) A concentration vs. relaxivity plot of nanocarriers incubated with cancer cells, with the y-axis representing the estimated concentration of internalized nanocarriers.

Carbon-coated core–shell multifunctional SPION (MFCSNP)-based drug delivery nanocarriers (MFCSNPs-FA-CHI-5FU nanocarriers) targeting folic acid and chitosan (FA-CHI) have been produced [159]. In addition to serving as a site-specific drug carrier, the pH-responsive release of 5-FU from MFCSNPs-FA-CHI-5FU nanocarriers demonstrates the utility of folic acid in this context. It was also discovered that MFCSNPs-FA-CHI-5FU nanocarriers show potential as T2-weighted MR contrast agents, and their contrast augmentation (signal darkening) in MR imaging is promising. Results from in vitro cytotoxicity tests as well as confocal microscopy imaging showed that MFCSNPs-FA-CHI-5FU nanocarriers are selective for folate-receptor-positive cancer cells and may be internalized via accumulation in the cell cytoplasm. Preliminary biological examination further supports the hemocompatibility and biocompatibility of the nanocarriers, allowing these multifunctional magnetic fluorescent NPs to be used as novel targeted theranostic devices. The effectiveness of MFCSNPs-FA-CHI-5FU nanocarriers as contrast agents was investigated using magnetic resonance imaging. By decreasing the magnitude of the transverse relaxation time, it has been proven that magnetic NPs can attenuate MR signal strength. Figure 11 shows a T2-weighted magnetic resonance image.

In order to be useful in diagnostic imaging, bimodal magneto-fluorescent materials require surface-engineered nanoparticles with excellent biosafety, pronounced colloidal stability, large magnetic moments, and robust photoluminescence (PL) emission. For in vivo magneto-resonance and fluorescence dual-mode imaging of malignant tumors, Mohandes et al. proposed polymer-coated nanoparticles (PCNPs) based on manganese ferrites covered with a thin shell of nitrogen-doped carbon dots. Hybrid magneto-fluorescent nanoparticles may be easily synthesized by in situ thermolysis of metal oxalates and phenylenediamine in diphenyl ether [160]. After 60 min, the tumor may be seen clearly since the fluorescence intensity in the area containing the tumor tissue appears to rise with time. The remarkable efficacy of PCNPs as T2 contrast agents in clinical diagnosis has also been demonstrated by magnetic resonance imaging (MRI) of phantoms and animal instances. High-resolution MRI imaging of cardiomyocyte death in vivo has the potential to advance the discovery of effective new cardioprotective treatments. Thus, in vitro comparisons were made between the new nanoparticle AnxCLIO-Cy5.5 and annexin V-FITC for detecting apoptosis in cardiomyocytes, and both demonstrated a significant degree of colocalization [161]. Five mice were administered AnxCLIO-Cy5.5 and four were given CLIO-Cy5.5, both at a dosage of 2 mg Fe/kg, followed by temporary blockage of the left anterior descending (LAD) artery, and MRI was performed. In vivo measurements of MR signal strength and myocardial T2* were taken in hypokinetic areas of the LAD distribution. To back up the in vivo results, fluorescence imaging was performed outside of living organisms. Mice treated with AnxCLIO-Cy5.5 had substantially shorter myocardial T2*s (8.1 vs. 13.2 ms, P 0.01) and a greater fluorescent target-to-background ratio (2.1 vs. 1.1, P 0.01). One more study described the use of a magneto-fluorescent theranostic nanocomplex targeted to neutrophil gelatinase-associated lipocalin (NGAL) for imaging and treatment of pancreatic cancer [162]. The agent was made up of a nanoshell with a silica core and a gold shell. An outer layer of silica encased approved NIR (ICG) and MR (iron oxide) contrast agents. Antibodies attached to the outsides of the complexes helped direct the agents to cells that made too much NGAL. Nanocomplex sizes were changed to give a plasmon resonance at about 808 nm.

## 6. Magneto-Fluorescent Nanobioprobe for Positron Emission Tomography (PET) Imaging

It has gradually become apparent that the behavior of cells in vivo is significantly different from that observed in vitro, due to factors such as the presence of many cell types, complicated anatomical characteristics, fluid flow forces, and combinations of cytokines and chemokines. Hence, there is a pressing need to create and verify imaging methods permitting the study of cells in their natural environments. With the advent of new cell-tracking imaging techniques, cells delivered in animal models may be observed with an unparalleled level of detail and specificity. Nuclear-medicine imaging techniques, such as PET scans, are available. Radiotracers are radioactive substances used in nuclear medicine that are administered intravenously and at extremely low doses. In contrast to other types of imaging, PET scans may detect changes in metabolic processes and cellular activity. They may now be able to detect illness in its infancy. Radiotracers are taken up at a higher rate by diseased cells than by healthy ones. The term for these areas of high uptake is “hot spots”. A PET scanner is able to detect this radiation and provide pictures of the afflicted tissue. PET scan pictures are combined with CT-scan X-rays to create a PET/CT scan. PET imaging’s distinctive characteristics of very high sensitivity and precise determination of in vivo concentrations of radiotracers stem from the physics of the emission and the detection of the coinciding photons. In oncology, cardiology, and neurology, PET imaging has become an integral part of clinical practice. Preclinical research has also benefited greatly from PET imaging, especially when it comes to studying mouse models of illness and other small-animal models. However, PET imaging systems encounter a number of obstacles. Issues including integration with X-ray computed tomography and magnetic resonance imaging, as well as the basic trade-offs between resolution and noise, need to be addressed.

**Figure 12:**
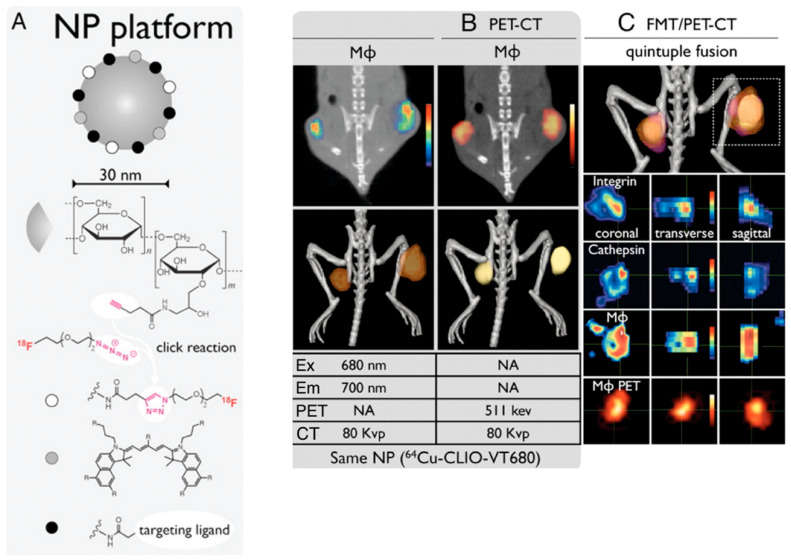
Nanoparticle-based multimodality PET imaging. (**A**) The broad range of targeted ligands that may be conjugated to CLIO, including, but not limited, to 18F through click chemistry and peptides. Tumor-bearing mice were coinjected with a fluorescent peptide against integrins, a fluorescent cathepsin sensor, and ^64^Cu-CLIO-VT680, and then subjected to in vivo multichannel PET-CT (**B**) and FMT/PET-CT (**C**) [163].

PET imaging can detect high-energy photons from decaying radioactive isotopes at low levels with far greater sensitivity than MRI. Nanoparticle labeling with PET isotopes such as ^18^F has effectively lowered the detection threshold and increased sensitivity [163]. At a clinically relevant dosage, a fluorescently derivatized CLIO was coupled with ^18^F by copper-catalyzed azide-alkyne cycloaddition, yielding a trimodal nanoparticle detectable through positron emission tomography (PET), fluorescence molecular tomography (FMT), and magnetic resonance imaging (MRI) (Figure 12). To put this another way, PET was 200 times more sensitive than MRI (16 times) and 50 times more sensitive than FMT (5 times) when it came to detecting 18F-magnetic nanoparticles. A strong positive correlation (r^2^ > 0.99) exists between PET and FMT signals in agar-based phantoms when CLIO-based particles are used that contain 18F and the fluorescent dye VT680.

**Figure 13:**
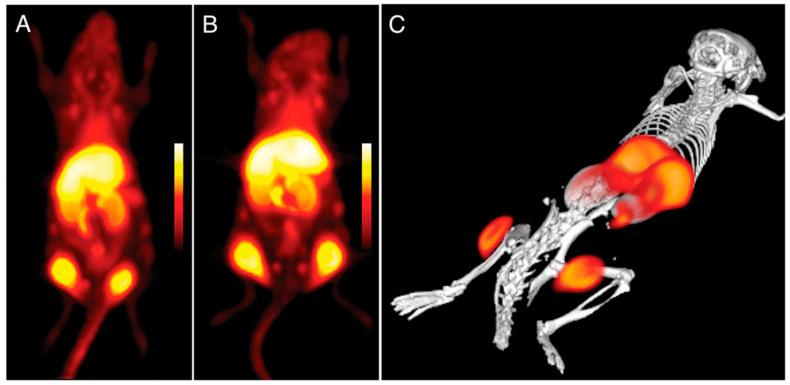
Imaging using 89Zr-labeled DNPs using PET in mice with bilateral flank tumors (24 h after administration). (**A**,**B**) Two distinct animals with almost identical tumor distributions (coronal stacks). Panel A depicts an animal; panel (**C**) depicts a three-dimensional model of the animal [167].

**Figure 14:**
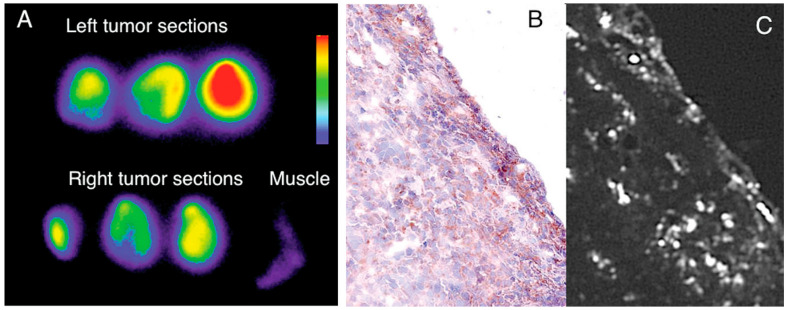
The study of DNP distribution using autoradiography and histopathology. Sections of tumor and adjacent normal tissue (**A**) autoradiographed to highlight predominant accumulations in malignancies. In (**B**), a typical Mac3 immunohistology specimen can be seen. The sections next to B show the distribution of fluorescent DNPs in Mac3-positive cells (**C**) [167].

Imaging using 18F-CLIO PET-CT revealed considerably increased PET signals in murine aortic aneurysms compared to normal aortas, likely because 18F-CLIO targeted monocytes and macrophages within the aneurysm [164]. Radionuclide, fluorescent, histologic, and flow cytometric tests all corroborated the cellular probe distribution. Presently, risk indicators, such as aneurysm diameter, gathered from the community are used to determine whether or not the surgical excision of an aneurysm is warranted. Therapeutic choices might be made on an individual basis using nanoparticle-PET agent conjugates in response to molecular illness indicators, such as cellular inflammatory activity in the aneurysm. Using PET for in vivo imaging of leukocytes enables researchers in the field of immunology to monitor the selective recruitment of particular immune cells at various points in the pathogenesis of an illness, locate infectious or inflammatory hotspots, and create evidence-based therapy plans [165]. The use of CNs for ^89^Zr leukocyte labeling for PET imaging of inflammation was described by Fairclough et al. [166]. The purpose of this research was to perfect the process of labeling leukocytes by enhancing the CN preparation for maximum ^89^Zr-loading and cell uptake. The effectiveness of cell uptake has been studied with respect to the nanoparticles’ size and surface charge. The absorption and retention of ^89^Zr-loaded CNs in mixed human leukocyte cells, as well as CNs’ affinity for ^89^Zr, have been studied. A novel technique for radiolabeling white blood cells with zirconium-89 (^89^Zr) or copper-64 (^64^Cu) for PET imaging was assessed. Ionotropic gelation was employed to create chitosan nanoparticles (CNs) that were then used to transport radiometals into white blood cells. Using the same ^89^Zr-labeled DNPs described previously, Keliher et al. performed PET-CT imaging in a mouse model of cancer. On account of its popularity for studying tumor infiltrating host cells, a syngeneic colon cancer (CT26) mouse model was employed [167]. Images from a variety of xenografted animals are shown in Figure 13. In certain animals, the concentration of RES in tumors was higher than in any other organ of the reticuloendothelial system, which was an unexpected finding. Through thorough correlative histology and flow cytometry experiments, we were able to ascertain whether tumoral accumulation was the result of cellular uptake or extraversion and interstitial accumulation. After removing tumors, we cut them into thin slices (1 mm) and used autoradiography to image them next to slides stained with Mac-3 (a stain that identifies macrophages). All in all, the “hot areas” and the Mac3 optimism across the chapters were in accord (Figure 13). Histological examination revealed selective uptake of fluorescent DNPs by Mac3+ cells and a general lack of absorption by Mac3 cells (Figure 14).

Mammary carcinoma is the second most frequent malignant tumor (after lung cancer) in adult women and the fifth leading cause of cancer-related mortality worldwide [168]. While existing clinical techniques may not be perfect, there is room for improvement in the diagnosis and monitoring of lymphatic involvement and recurrence. For the time being, the sentinel-lymph-node technique is the most reliable method for identifying nodal invasion in the lymphatic axillary chain. This method, however, is fairly intrusive, since it relies on a surgical biopsy of the first lymph node of the axillary chain detected using lymph scintigraphy with ^99m^Tc [169]. After designing and testing a wide variety of bifunctional magneto-fluorescent nanoparticle systems (MFNs), Corsi et al. narrowed down to one MFN type that met the aforementioned requirements with respect to sensitivity, in vitro safety, and physiological behavior. Accordingly, the MFN-based multifunctional contrast agent we disclose here appears to possess all the necessary features for its future development for the in vitro and in vivo imaging of early mammary cancer. Size, shape, zeta potential, fluorescence intensity, T_2_ relaxivity enhancement in water protons, and stability are all factors in determining the quality of these nanoparticles. Therefore, two were created, and their internalization processes, intracellular destinies, and toxicities in MCF-7 cancer cells were investigated.

## 7. Quantum Dot-Magnetic Nanoparticle Assembly as a Site-Specific Imaging Probe

**Figure 15:**
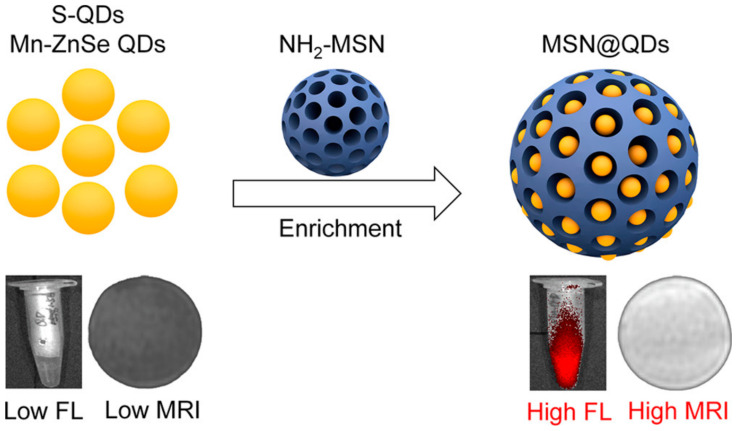
Improved Dual-Modal Imaging Through Enrichment Process Schematic [176].

**Figure 16:**
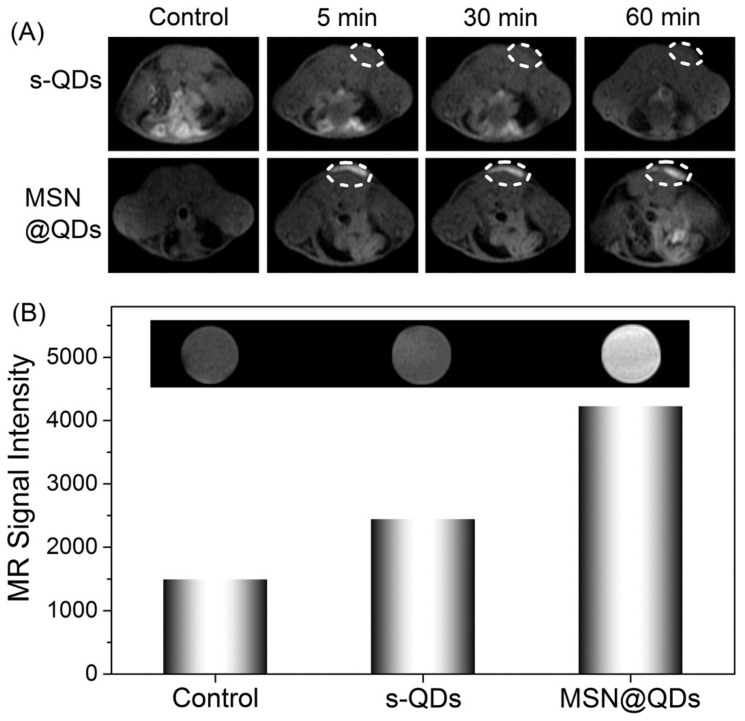
Comparison of pre- and post-injection MR images of a Balb/c mouse injected with s-QDs and MSN@QDs: MR coronal pictures at T1 weighting (**A**) and the associated MR signal intensity (**B**) as measured in vitro [176].

Since their beginnings, nanoscience and nanotechnology have revolved around the concept of fluorescent quantum dots (QDs), which are typically described as having a diameter size of 10 nm. Several distinct structural, electrochemical, and photochemical features of QDs make them potentially useful platforms for sensing applications. Nanocrystals of semiconductor elements from groups II–VI, III–V, and IV are commonly referred to as quantum dots because of their luminous properties [170]. Their sub-10 nanometer dimensions provide them with unique optical and electrical characteristics compared to those of bulk materials. These 1D nanomaterials are amenable to surface changes for added functionality. Some QDs have reactive functional groups, such as amines, carboxylic acids, alcohols, and thiols, in their coats because they are stabilized in aqueous solutions. A simple method was provided for producing quantum dots (QDs) magnetically doped with Fe with a size range of 3–6 nm that are water-dispersible and emit in the near-infrared (NIR). In situ hydrothermal doping of alloyed CdTeS nanocrystals with Fe was used to achieve this [171]. Using N-acetyl-cysteine (NAC) ligands, which have both thiol and carboxylic acid functional groups, anchored magnetic quantum dots (MQDs) showed high saturation magnetization (85 emu g^−1^). These NPs could also emit fluorescence in the NIR range and had a proton transverse relaxivity of 3.6 mM^−1^ s^−1^. Phantom and in vitro investigations have shown that NIR MQD functionality may be evaluated. These unaggregated Fe-doped CdTeS MQDs have the potential to operate as multimodal contrast agents for tracking living cells due to their water-dispersibility, NIR emission, and MR contrast. Using the free-radical polymerization approach, nanocomposites consisting of ZnS:Mn2+ quantum dots (QDs) and Fe_3_O_4_ QDs/SiO_2_/P(NIPAAm-co-AAm) core–shell-shell structures have been effectively synthesized [172]. HeLa cell cytoplasm fluoresces red, demonstrating its usefulness for biolabeling. Citric acid and manganese tetraphenyl porphyrin were used as carbon sources in an aqueous medium to create manganese-doped carbon quantum dots (MnCQDs) in a single hydrothermal process [173]. X-ray photoelectron spectroscopy, transmission electron microscopy, and diffraction confirmed the MnCQD structures. Under UV irradiation (365 nm), MnCQDs emit strong green luminescence with a peak at 482 nm and a fluorescence quantum yield of 13%. At a detection limit of 220 nM, ferric ion in aqueous solution may be detected with the use of MnCQDs as a fluorescent probe. MnCQDs were shown to have little cytotoxicity in an MTT experiment using HeLa cells. In MnCQDs, the presence of paramagnetic ions results in an improved magnetic resonance (MR) signal. Therefore, MnCQDs can function as good MRI contrast agents. A nanoprobe for fluorescence and magnetic resonance (MR) imaging synthesized with ease was described by shi et al. via a method in which Gd3+ ions are chelated onto the surface of cDTPAA-functionalized carbon quantum dots. This allows the nanoprobe to have bright fluorescence and significantly improved relaxivity (CQDs) [174]. The CQD–DTPA–Gd nanoprobe that was created as a consequence has exceptional water solubility, great fluorescence efficiency, exceptionally high relaxivity, and almost no cytotoxicity. Superparamagnetic nitrogen-doped carbon-iron oxide hybrid quantum dots (C-Fe_3_O_4_ QDs) might also be employed for FL/MR/CT bioimaging [175]. For example, C-Fe_3_O_4_ QDs are produced by a green and straightforward one-pot hydrothermal technique that employs poly(-glutamic acid) as a precursor and stabilizer. As-prepared C-Fe_3_O_4_ QDs have high water miscibility, spectral FL properties with high quantum yields of 21.6%, exceptional photostability, robust superparamagnetic characteristics, and cytocompatibility. Additional proof that as-prepared C-Fe_3_O_4_ QDs are ready for use in FL/MR/CT triple-modal tumor imaging came from in vivo bioimaging of tumor-bearing nude mice, which combined FL, MR, and CT images. Mn-doped quantum dots (QDs), and in particular Mn-doped ZnS (ZnSe) QDs, are appealing for fluorescence/magnetic resonance imaging (MRI) dual-mode imaging because of their distinctive fluorescent and magnetic characteristics [176]. However, for MRI imaging, a minimal concentration of dopant (Mn^2+^) is sufficient to maximize fluorescence in QDs. Here, an enrichment technique with mesoporous silica (MSN) loading (Figure 15) was investigated for the purpose of producing a highly luminescent/paramagnetic Mn-doped ZnSe QD assembly (MSN@QD) for enhanced MRI/optical dual-model imaging. The QD loading density in MSNs was calculated to be 152 ± 12. The MSN@QD assembly fluorescence was enriched with QDs (enrichment factor = 143) upon loading. No significant concentration quenching was seen, despite the huge Stokes shift (200 nm). Enhanced local Mn^2+^ concentration also led to a boost in T_1_ MR contrast (Figure 16), allowing for signal enrichment to be achieved in magnetic resonance imaging (MRI).

A carbon quantum dot (CQD)-stabilized gadolinium hybrid nanoprobe (Gd-CQD) was described by Xu et al., which was made by hydrothermally treating citrate acid, ethanediamine, and GdCl_3_ in one pot at 200 °C for 4 h [113]. High biocompatibility and minimal toxicity were proven by in vitro and in vivo testing. Due to their high Gd contents and hydrophilicity, the Gd-CQDs were shown to have a greater MR response than gadopentetic acid dimeglumine (Gd-DTPA). Finally, Gd-CQDs retained their CQD-derived fluorescence. Zebrafish embryos and mice were used to verify that Gd-CQDs may be imaged in vivo using both magnetic resonance (MR) and fluorescence. High affinity for U87 cancer cells was achieved by targeted imaging using Gd-CQDs modified with arginine-glycine-aspartic acid (RGD) tripeptide. In order to create multimodal nanoprobes that make use of both optical and magnetic imaging, gadolinium (Gd) complexes containing CDs were generated by a one-step microwave technique [177]. The generated Gd-CDs showed strong fluorescence and had great water solubility and biocompatibility. It was shown that apoferritin (AFn) nanocages labelled with Gd-CD compounds might serve as good T_1_ contrast agents for magnetic resonance imaging because Gd-doped CDs dramatically improved the circulation duration and lowered the toxicity of Gd^3+^ in in vitro and in vivo magnetic resonance imaging. Cancer theranostics is a promising area for the application of self-assembling multifunctional Gd-CDs/AFn (DOX)/FA nanoparticles. When designing multifunctional nanostructures for use in biological imaging, it is preferable to include luminescent imaging agents and MRI contrast agents. Fluorescence microscopy and magnetic resonance imaging (MRI) may both make use of luminescent biocompatible silicon quantum dots (SiQDs). This is the first report of the production of a nanocomplex including SiQDs and gadolinium ions (Gd^3+^) for use in biology [178]. The optical characteristics of the probes were confirmed to be unaltered after they were taken up by cells and transported into the intracellular space. It was found that the nanostructures had a magnetic resonance relaxivity of 2.4 mM^−1^ s^−1^ (in terms of Gd^3+^ concentration), which works out at around 6000 mM^−1^ s^−1^ per nanoconstruct. The newly developed probe’s appealing optical and relaxivity characteristics pave the way for SiQDs to be used in future multimodal applications, such as cancer imaging. The virtues of nanoscale ternary chalcogenides, including their adaptability and wide range of energy and biomedical uses, have resulted in a surge in their study. Aqueous synthesis of silver indium sulfide quantum dots with dual ligands of glutathione and polyethyleneimine has been reported [179]. The resulting silver indium sulfide quantum dots have a long lifespan of 3.69 s, great fluorescence stability, and minimal cytotoxicity, making them a promising tool for live-cell imaging. Since it offers a practical and novel technique to control the inherent characteristics of carbon quantum dots (CQDs) and graphene quantum dots (GQDs), doping fluorescent carbon dots (DFCDs) with heteroatoms has lately gained a lot of interest in comparison to conventional fluorescent materials.

**Figure 17:**
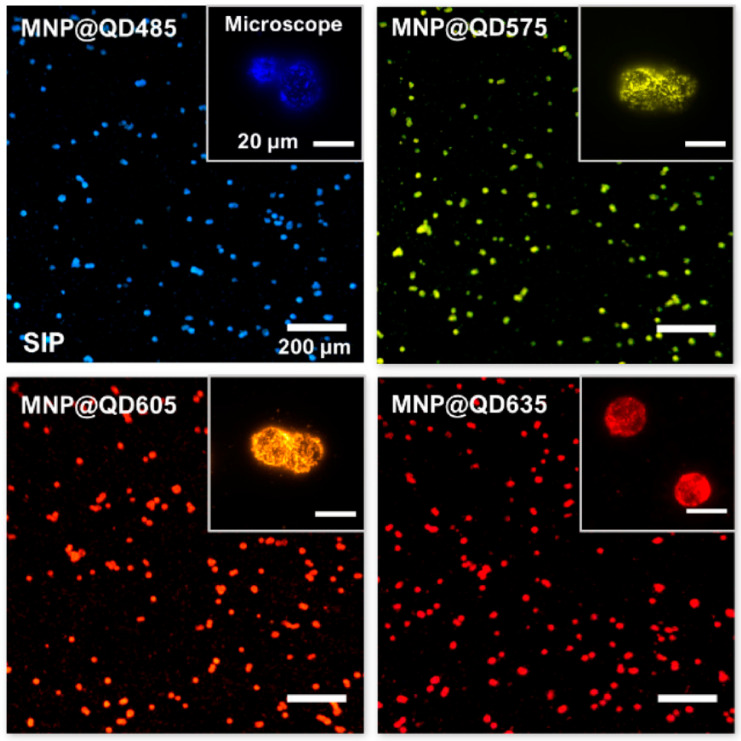
Smartphone (main images, scale bar = 200 m) and microscope (insets, scale bar = 20 m) images of fixed SK-BR3 cells isolated with MNP@QDs of different colors: QD485, QD575, QD605, and QD635. The notation for the QDs indicates the QDs’ peak PL emission wavelengths. The smartphone pictures were taken in RGB color format. The microscope images were given “false colors” based on the monochrome intensity values [183].

The photostability and luminosity of QDs can be greatly improved by including them in nano- or micromatrix composites [180]. Putting quantum dots and other functional nanoparticles in the same matrix is predicted to provide imaging probes that can detect many modalities. Making nanoprobes out of common nanomaterials (such as quantum dots, gold nanoparticles, and iron oxide nanoparticles) is a crucial step towards improving in vivo multimodality imaging. Strong processes and low-toxicity synthetic techniques for multimodality nanoprobes are urgently needed in nanomedicine domains [181]. Using magnetic nanoparticles (high T2 relaxivity), visible-light-emitting CdSe/ZnS quantum dots (QDs; Em = 600 nm), and near-infrared (NIR)-emitting CdSeTe/CdS QDs, MA et al. developed a simple and robust technique for developing multilayered nanoparticles (MQQ-probe) (Em 780 nm) [182]. A variety of nanoparticles were coated in layers of silica. Core–shell nanoprobes have many layers, each of which can give a unique set of features depending on the nanoparticles they contain. In order to facilitate easy, quick, and selective cell extraction, fluorescent labeling, and counting, Tran et al. demonstrated a prototype smartphone-based imaging platform (SIP) coupled with magneto-luminescent suprananoparticle assemblies [183]. As shown in Figure 17, the author provided several instances of high-magnification photographs of the separated cells that were taken with a microscope designed for scientific study. For the four colors of the MNP@QDs, the signal-to-noise ratios (SNRs) on the SIP were 7 3 (SD), 8 3, 14 5, and 9 3 for cells labelled with MNP@QD485, MNP@QD575, MNP@QD605, and MNP@QD635, respectively. The production of these suprananoparticle assemblies and their immunoconjugates by self-assembly is extremely beneficial, and the ultrabright PL of these assemblies makes it possible to image single cells while maintaining a high signal-to-noise ratio. Proof of concept was achieved by isolating and counting HER2-positive SK-BR3 breast cancer cells against a background of HER2-negative MBA-MD-231 breast cancer cells. This allowed for comparison of the two populations. The extraordinary physical and chemical capabilities of inorganic nanoparticles have led to their introduction into biological systems as effective probes for in vitro diagnostics and in vivo imaging. To combine the strengths of MR and fluorescence imaging, a novel class of color-tunable quantum dots (QDs) called Gd-Zn-Cu-In-S/ZnSs (GZCIS/ZnSs) have been synthesized and put to use [184]. By integrating Gd into ZCIS/ZnS QDs, we were able to successfully fabricate GZCIS/ZnS QDs with significant MR enhancement and without sacrificing the fluorescence characteristics of the original ZCIS/ZnS QDs. High PL quantum yield and “color-tunable” PL emission from 550 to 725 nm may be achieved by varying the Zn/Cu feeding ratio in the as-prepared GZCIS/ZnS QDs (QY). A bovine serum albumin (BSA) coating was used to introduce the GZCIS/ZnS QDs into water.

## 8. Conclusions and Perspectives

This review was motivated by the plethora of ground-breaking studies conducted all across the world. Insight into the manufacturing of fluorescent NPs and the methods for imbuing them with magnetic properties may be gained from this debate. There are extremely high requirements for and interest in these resources. Based on what has been said, it is evident that magneto-fluorescent nanocomposites provide novel avenues for research in chemistry, biology, and medicine. Despite recent advancements, the field of fluorescent-magnetic nanocomposite materials is still in its infancy, and much work needs to be done to further improve these materials and their applications. Magneto-fluorescent hybrids of varying sizes, architectures, dispersions, and surface modifications have been synthesized using the various approaches discussed here. In point of fact, surface modifications of these nanoparticles with other compounds, polymers, and silica lead to the stabilization of the nanosystems as well as decrease in toxicity, which allows for biological applications. In spite of the fact that toxicological studies have been conducted on some of these NPs, the precise mechanism by which nanoparticles cause toxicity is still unknown. As a result, a more in-depth investigation into the toxicity and biocompatibility of fluorescent-magneto hybrid NPs and the constituents that make them up is required. Significant progress has been achieved in the synthesis of nanoparticles with adaptable designs, paving the way for both intelligent delivery and individualized therapy. Nanoparticles with many functions, such as magneto-fluorescent nanoparticles, are without a doubt the most helpful instruments now available. Although there is an immediate need for various modifications and new insights into the manufacturing of magneto-fluorescent nanoparticles and their toxicities, these particles show significant promise for use in medical diagnosis and other applications. Recently, several kinds of luminous materials, including semiconductor quantum dots (QDs) and organic urophores, have been linked with magnetic units to produce MFNPs. These MFNPs may operate both as optical and magnetic probes due to the coupling of the two types of materials. The fact that these QDs often include heavy metals, such as cadmium, lead, and other substances, which have the potential to be poisonous, have lower water solubility, and pose environmental risks limits the range of uses for these materials.

## 9. Present Market and Future Development

Magnetic particle imaging (MPI) is a novel imaging technique that, like MRI and positron emission tomography, has the potential for high-resolution imaging without requiring any intrusive procedures (PET). It is anticipated that MPI will be able to follow cells for cancer-therapy monitoring. A possibility exists that CAR T cells will take up SPIONs. SPIONs may be taken up by macrophages in active phagocytic sites or by atherosclerotic plaques if they are delivered systemically [185]. In a magnetic particle imager, the signals produced by SPIONs of different sorts and with varied features, such as core size, or by the same SPIONs in different settings can be easily distinguished. Both bound and unbound SPIONs provide distinguishable signals which can prove useful in preclinical research. The variations in the produced signals may be used to create multicolored pictures, with each hue representing a unique SPION or a SPION in a distinct environment [186]. The amplifier noise level can be lowered to the same level as the patient noise contribution via processes such as cryogenic cooling of the amplifiers and adjusted tuning. Industrial efforts to create an MPI/MRI scanner which combines the benefits of these two imaging modalities are quite promising [187]. One of the pillars of future technologies that will allow for the development of superior systems with high spatial and temporal resolution is the creation of multimodal imaging systems that use fluorescence imaging. Ongoing research and development of new algorithms is also contributing to overcoming the shortcomings of present imaging systems, particularly with respect to depth of penetration. Fluorescence imaging has become a widely utilized and increasingly popular tool in preclinical biomedical research as a result of its sensitivity, cost-effectiveness, and safety, as well as the availability of a wide range of functionalized fluorophores. Possible future applications of fluorescence imaging in clinical medicine may be seen in its current applications, such as cancer detection and therapy and surgical planning. Fluorescence imaging is projected to play a larger role in the clinical care of patients as technology improves, opening the door to imaging-driven tailored therapy. Cost estimates were derived from an analysis of publicly accessible financial data from the annual reports of major firms producing and selling imaging agents. We compared these figures with the extensive data and analysis we have on the price of creating medicinal medications. When compared to the current revenues of blockbuster imaging medicines, which are between USD 200 and 400 million, it is clear that the development and commercialization of a drug for diagnostic imaging is rather expensive. All of the top-selling radiological agents have been available for quite some time. The market size for imaging agents is substantially smaller than that for therapeutic medications. In 2004, sales in the United States, which account for over half of global sales, amounted to USD 2.8 billion [188]. From a commercial perspective, there are five primary manufacturers of imaging agents. Among them are GE, BMS, Bracco, Schering, and Tyco, which were formerly a part of Amersham. While researchers try to perfect magnetic scanning devices, others refine nanoparticles for injection into patients or for the delivery of medications. The ultimate goal is to create universally effective nanoparticles that can cross biological barriers that are not homogeneous [189]. The use of artificial intelligence (AI) in radiography is a hotly debated issue at the moment. Machine learning, representation learning, and deep learning are the three primary subfields in artificial intelligence [190]. The fields of deep learning and machine learning are the most applicable to radiology. Inevitably, as time goes on, many of the existing technological flaws will be fixed, allowing for the creation of a highly sensitive and secure imaging system for medical diagnosis, which is desperately needed to satisfy societal expectations.

## Figures and Tables

**Table 1:** Synthetic tailored magneto-fluorescent multimodal nanoparticles (NPs) and potential uses.

Nanoparticles	Method	Targeting Ligand	Application	Ref.
Fe_3_O_4_/anti-IgG/GQD/BSA	Coupling	Human IgG	Urine renal disease	[83]
Ab (anti-aflatoxin B1)–CdS–Fe_3_O_4_ bioconjugates	Coupling	Aflatoxin B1	Detection of aflatoxin B_1_ B_1_ in corn samples	[84]
Dox-loaded carbon dot (CD)–4-carboxyphenylboronic acid (CBBA)–MnFe_2_O_4_ NPs [DCCM]	Coupling	Sialic acid	HeLa cells	[85]
Iron oxide superparamagnetic NPs-PEG-Cypher5E/folic acid	Coupling	Folic acid	MR imaging and fluorescence imaging	[86]
Fe_3_O_4_(MNP)-Cds(QDs)-folic acid	Coupling	Folic acid	As a delivery agent and an in vitro imaging diagnostic agent	[87]
MTX-PEG-CS-IONPs-Cy5.5	Coupling	Folic acid	Dual-model imaging and synergistically self-targeted cancer therapy	[88]
Fe_3_O_4_-dopamine hydrobromide (DPA)-PEG-FA/FITC NPs	Coupling	Folic acid	Targeted imaging of various tumors	[89]
Fe_3_O_4_-CdTe-humanized monoclonal antibody CC49 (hCC49 antibody)	Coupling	Tumor-associated glycoprotein-72 (TAG-72)	Cancer cell imaging	[90]
Fe_3_O_4_@mSiO_2_–triphenylphospine (TPP)/CD	Coupling	Mitochondria	Mitochondrial diseases	[91]
BRCAA1 antibody-FMNPs (Fe_3_O_4_-CdTe)	Encapsulation	BRCAA1 protein	In vivo dual-model imaging of gastric cancer	[92]
FMN (flavin mononucleotide)-coated ultrasmall superparamagnetic iron oxide (FLUSPIO)	Encapsulation	Riboflavin (Rf)	Prostate cancer xenografts	[93]
MNPs@OPE[oligo(*p*-phenylene ethynylene)]-PEG-FA		Folate receptor	Targeted magnetic resonance and two-photon optical imaging in vitro and in vivo	[94]
Fe_3_O_4_@SiO_2_/RhBITC-anti-HER2 antibody NPs	Encapsulation	Human epidermal growth factor receptor 2 (HER2)	Discrimination of HER2-positive breast cancer cells	[95]
Fe_3_O_4_@SiO_2_(FITC)-FA/AICAR(5-aminoimidazole-4-carboxamide-1-β-D-ribofuranoside)/DOX	Encapsulation	Folate receptor	Inhibition of cancer cell growth	[96]
PTX/Fe_3_O_4_ NPs/CuInS_2_/ZnS QDs@biotin–PEG–PCD [abiotin–poly(ethylene glycol)–poly (curcumin-dithiodipropionic acid) copolymer]	Encapsulation	Biotin receptor	Treatment of multidrug-resistant breast cancer at the cellular level	[97]
Trastuzumab-conjugated Lipo[MNP@m-SiO_2_(FITC)]	Encapsulation	Her2/neu	In vitro fluorescence and MR imaging of Her2/neu-positive breast cancer	[98]
Fe_3_O_4_/CuInS_2_(CIS)@SiO_2_(Gd–DTPA)–RGD (arginine-glycine-aspartic acid)	Encapsulation	αVβ3 integrin	MR and fluorescence imaging of pancreatic cancer	[99]

**Table 2:** Different types of magneto-fluorescent nanoparticles, their magnetic characters, and specific applications.

Magnetic Part	Fluorescent Part	Size (nm)	Magnetization (emu/g)	Application	Ref.
Fe_3_O_4_	FITC	125	-	Cell imaging	[126]
Fe_3_O_4_	RhB	30	8	Cell membrane imaging	[127]
CoFe_2_O_4_–Cr_2_O_3_	-	30	5	Cell imaging	[128]
γ-Fe_2_O_3_	FITC	9	-	MRI and fluorescence imaging	[129]
Fe_3_O_4_	Poly(methacrylic acid)	280	30–60	Cell labeling and drug delivery	[130]
Fe_3_O_4_	CdSe/CdS QDs	9	15	Mouse brain imaging	[131]
Fe_3_O_4_	RITC/SiO_2_	60	-	MRI and fluorescence imaging (tumor)	[132]
Fe_3_O_4_	QDs/PEG	150	-	Circulatory fluorescence imaging	[133]
Fe_3_O_4_	CdSe/ZnS	35–45	-	Cell imaging	[134]
Fe_3_O_4_	CdTe/ZnS	185	37	Anticancer drug release and imaging	[135]
Fe_3_O_4_	FITC	11	-	Fluorescence imaging	[136]
Fe_3_O_4_/Fe_2_O_3_	Cy5.5	97	-	Neovasculature	[137]
Fe_3_O_4_	Yb^3+^/Er^3+^/Tm^3+^/NaYF_4_	80	38	Cell imaging	[138]
Fe_3_O_4_	CdSe	-	-	Cell imaging	[139]
Fe_3_O_4_	RhB	-	-	Cell imaging	[140]
Fe_3_O_4_	CdTe	70–80	6	Cell imaging and drug delivery	[141]
Fe_3_O_4_	CdTe	50–1000	-	Field-assisted cell alignment	[142]
Fe_3_O_4_	CdTe	34	60	Cell imaging	[143]
Fe_3_O_4_	FITC	14	55	MRI contrast agent	[121]
Fe_3_O_4_	Atto 390/fluorescein/Rh6G	100–400	-	Magneto-sensitive fluorescence imaging	[144]
Fe_3_O_4_	Ce6	20–30	50	Tumor cell imaging	[145]
CoFe_2_O_4_	RhB/RITC	60	-	Cell imaging	[146]
FePt	CdS	10	5	Cell imaging	[147]
Fe_3_O_4_	FITC	600–700	17	Cell imaging	[148]
Fe_3_O_4_	ZnS	100	30	Cell imaging	[149]
Fe_3_O_4_	CdTe/CdS	8	-	Cell imaging	[150]
Zn_0.4_Fe_2.6_O_4_	Drug	160	-	Drug release and imaging	[151]
FePt	Atto 590	5	-	Cell imaging	[152]
Iron oxide	Cy7	21	-	MRI	[153]
Fe_3_O_4_	PDI-PAA	60	7	Cell imaging	[154]
Fe_3_O_4_	FITC	36	23	Cell imaging	[155]
Fe_3_O_4_	Squarylium indocyanine	51	8	Cell imaging	[24]

## Data Availability

Not applicable.
